# The management of thoracolumbar burst fractures: a prospective study between conservative management, traditional open spinal surgery and minimally interventional spinal surgery

**DOI:** 10.1186/s40064-015-0960-4

**Published:** 2015-04-30

**Authors:** Amit Kumar, Randeep Aujla, Christopher Lee

**Affiliations:** Specialist Orthopaedic Registrar, University Hospitals Leicester, Infirmary Square, Leicester, LE1 5WW UK; Consultant Orthopaedic & Spinal Surgeon, Lincoln County Hospital, Greetwell Road, Lincoln, LN2 5QY UK

**Keywords:** Thoracolumbar fracture, Minimally invasive surgery, Open surgery, Oswestry disability index, Single level

## Abstract

The objective of this study was to assess which patient group had better outcomes for management of single level thoracolumbar spinal fractures. We prospectively collected data on the outcomes of patients having either conservatively managed, traditional open surgery, or minimally interventional surgery (MIS) for treatment of a single level thoracolumbar fracture. All patients had previously asymptomatic spines prior to their fractures and had a single level thoracolumbar burst fracture of more than 20° kyphosis. Fractures treated operatively, either via open surgery or MIS techniques, were corrected to less than 10° of residual kyphosis using a monoaxial pedicle screw construct 2 levels above & 2 levels below the fracture posteriorly only. The metalwork was removed between 6 months and 1 year post operatively to remobilise the spinal segments. All patients were then evaluated at least 6 months after metal work removal and at 18 months post fracture using radiographs and the Oswestry Disability Index (ODI).

Those patients treated with MIS techniques demonstrated superior outcomes compared to traditional open techniques and conservative methods of treatment, with significantly reduced hospital stay, better return to work & leisure, and the best chance of restoring their spine to near its pre-injury status. We would recommend MIS techniques as the best way of treating single level thoracolumbar spinal fractures. There is a significant improvement in ODI when treated by MIS over open surgical methods.

## Introduction

The treatment of thoracolumbar burst fractures of the spine still excites debate and disagreement. In patients without neurological deficit, there are those who advocate conservative treatment whatever the instability or deformity (Chow et al. 1996; Shen et al. 2001; Cantor et al. 1993). However, some patients are left with significant disability when fractures have healed with a significant deformity, particularly kyphosis (Xiang-Wang et al. 2008; Shen and Shen 1999). Many would advise fixation and correction of fractures with kyphosis more than 30°, and a significant number with deformity less than that (Xiang-Wang et al. 2008; Shen and Shen 1999; Kim et al. 2011; Logroscino et al. 2009; Tezeren et al. 2009). The risk of late collapse in conservatively managed patients is a risk. Spinal braces are poor at preventing this and fixation usually avoids this problem (McAfee et al. 1982). There are many that claim that operative fixation carries a morbidity risk, with significant risk to soft tissues, particularly paraspinal muscles (Kim et al. 2009; MacNab et al. 1977). Fixation also carries the risk of failure of correction and loss of position, particularly with older types of implants and short segment fixation (Xiang-Wang et al. 2008; Alanay et al. 2001; Tezeren and Kuru 2005).

Minimally interventional surgery (MIS) seeks to avoid the soft tissue damage that comes with traditional open techniques, and allows the benefits of longer constructs (Logroscino et al. 2009; Tezeren and Kuru 2005; Choll 2010; Ringel et al. 2006; Hatta et al. 2009; Smith et al. 2010). MIS techniques, however, have to deliver correction and stabilisation that is as good as with conventional open techniques, without compromise to implant placement or increased risk of complications, and allow easy implant insertion and removal. Such techniques involve placing pedicle screws through small paraspinal incisions, preserving the overlying muscles and soft tissues, then sliding a rod bent to the appropriate shape under the muscles from one incision to the other before locking it down into the screws with the appropriate end-caps, reducing and stabilising the fracture.

To date there have been no prospective trials that demonstrate the benefits of MIS techniques over others when correcting or stabilising spinal fractures and none that directly compares open and MIS techniques using exactly the same implants for both. Our study looks at prospectively collected data of the past nine years, which addresses this.

## Results

In total 78 patients met the inclusion criteria for the study. 30 patients were treated conservatively, 23 patients were treated via open operative techniques, and 25 patients were treated via MIS techniques. The total cohort confirmed a pre-injury ODI score of 0, and had a single level spinal fracture at T12, L1 or L2, with a local kyphosis greater than 20°. There were 29 fractures (37%) involving T12, 41 fractures (53%) involving L1, and 8 fractures (10%) involved L2. The youngest was 18 years and oldest was 53 years of age at the time of injury. There was no variance in patient demographics or characteristics between conservative, open conventional surgery or MIS groups (Table 1). The mechanism of injury and fracture characteristics are shown in Table 2 and 3 respectively.

**Table 1:** **Demographics and patient characteristics for the three treatments groups for single level spinal fractures**

**Demographics**	**Conservative (%)**	**Open surgery (%)**	**MIS (%)**
Number of patients	30	23	25
Mean age (range)	31 (21–52)	29 (19–49)	31 (18–53)
Gender M:F	19:11	15:8	14:11
Manual occupation	12 (40)	10 (44)	11 (44)
Non-manual occupation	16 (53)	11 (47)	12 (48)
Sports person	2 (7)	2 (9)	2 (8)

**Table 2:** **Incidences of different mechanism/place of injuries for the spinal fractures treated conservatively or by different surgical methods**

**Mechanism of injury**	**Conservative (%)**	**Open surgery (%)**	**MIS (%)**
Road traffic accident	12 (40)	10 (44)	11 (44)
Sporting injury	8 (27)	5 (22)	6 (24)
Industrial injury	4 (13)	4 (17)	4 (16)
Domestic injury	6 (20)	4 (17)	4 (16)

**Table 3:** **Fracture characteristics for the spinal fracture groups treated conservatively or by different surgical methods**

**Fracture characteristics**	**Conservative (%)**	**Open surgery (%)**	**MIS (%)**
T12	12 (40)	9 (39)	8 (32)
L1	15 (50)	12 (52)	14 (56)
L2	3 (10)	2 (9)	3 (12)
Magerl Type A	19 (63)	9 (39)	11 (44)
Magerl Type B	11 (37)	14 (61)	14 (56)
Mean Post-Traumatic Kyphosis (degrees)	24 (20–27)	26 (20–33)	26 (21–34)

Analysis of data showed there was no difference in degree of post-traumatic kyphosis between the groups (p = 0.79). There is a significant difference in the time spent in hospital between conservative treatment (mean 36 days) and any surgical intervention (mean 2–4 days, p < 0.005). There is also a significant difference in time spent in hospital between the two surgical groups, with favourable results for MIS (mean 4 days for open surgery versus mean 2 days for MIS; p < 0.001, Table 4).

**Table 4:** **Time spent in hospital in days, time taken to return to work in months, and ODI at all time scales**

	**Conservative (Range)**	**Open surgery (Range)**	**MIS (Range)**
Time in hospital (days)	36 (10–104)	4 (2–7)	2 (1–4)
Time to return to work (months)	9 (3–24)	4 (0.5-9)	2 (0.1-6)
ODI prior to metalwork removal	n/a	14 (4–26)	4 (0–10)
ODI at 18 months	32 (12–46)	14 (4–26)	4 (0–10)
ODI at 30 months	32 (12–46)	14 (4–26)	4 (0–10)

In the conservatively managed cohort 8 patients (27%) did not return to their original occupations. 5 (17%) of these eventually returned to a less demanding occupation, and 3 (10%) became unemployed. In the conventional surgery group, 4 (17%) patients did not return to original occupation and eventually returned to a less demanding occupation. For the MIS cohort all patients returned to their original occupations.

With regard to the ODI, our results showed that there is a significant difference between MIS (ODI = 4) and conventional open treatment (ODI = 14) at all ODI time scales (p < 0.0001). There is an even greater difference between MIS (ODI = 4) and conservatively treated patients (ODI = 32) (p < 0.0001) for both 18 and 30 months. At 30 months follow up the ODI scores failed to improve and were unchanged in all groups (Table 4). The MCID for the ODI at 30 months for conservative, conventional surgery and MIS are 4.5, 2.8 and 1.7 respectively.

**Figure 1:**
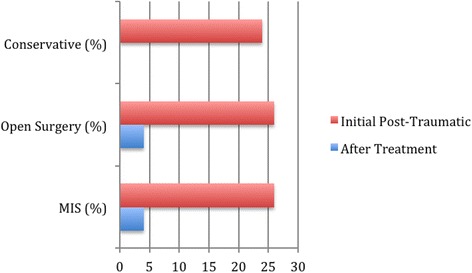
Chart showing mean kyphosis (degrees) post treatment.

The degree of correction achieved using the two surgical techniques showed no significant difference (p = 0.8). Patients whom underwent conventional open surgery had a mean kyphosis of 3.5° after correction. This was an 87% improvement on their initial post traumatic kyphosis (see Table 5 and Figure 1). The MIS cohort had a mean corrected kyphosis of 3.7°, giving an 86% improvement. The residual kyphosis after removal of metalwork remained the same as after fixation.

**Table 5:** **Showing the degree of kyphosis pre and post treatment**

**Fracture kyphosis**	**Conservative**	**Open surgery**	**MIS**
**Mean in degrees**			
**(Range)**			
Initial post- traumatic	24 (20–27)	26 (20–33)	26 (21–34)
Post treatment	25 (20–32)	4 (0–8)	4 (0–7)
Initial post- traumatic Magerl Type A	23	22	23
Post treatment Magerl Type A	23	2	3
Initial post- traumatic Magerl Type B	25	28	28
Post treatment Magerl Type B	25	4	4

There was no significant difference in improvement in kyphosis of patients with Magerl Type A (p = 0.6) or Type B (p = 0.4) fractures between the Open surgery group and MIS group. There was no significant change in kyphosis in those patients managed conservatively from post-traumatic to final degree of kyphosis (P > 0.5).

The mean operative time for both surgical groups (conventional open and MIS) was 120 minutes. The mean blood loss was 80mls for the MIS group and 550mls for the conventional open group. No patients required blood transfusion post operatively in either surgical group.

### Complications

In the conservative group, 6 patients had a kyphosis that deteriorated further (mean 5°, 3-8°) during the follow-up period with 5 patients requiring subsequent procedures to correct post-traumatic kyphosis. These patients underwent anterior reconstructive techniques to restore stability and kyphosis. The other complication that did exist in 4 patients was a transient bowel ileus. All cases resolved spontaneously without treatment and its occurrence likely to be related to bed rest. Only one patient had loss of correction (3°) in conventional open operative group and one patient had a post-operative wound infection that settled with antibiotics. There were no complications in the MIS group.

There were no cases of venous-thromboembolism (DVT or PE). All patients received mechanical prophylaxis and when deemed safe commenced on low molecular weight heparin after 5 days while recumbent. Patients had mechanical prophylaxis also during surgery and were mobilised post-operatively as soon as safely possible. This was a universal departmental policy.

Patients treated with bed rest had regular pressure areas checks and log-rolls to avoid decubitus ulcers. There were no incidences of other medical complications such as chest infection or respiratory problems. We feel our incidence of VTE was low as the cohort consisted of young fit patients with little other medical co-morbidities and the spine injury as their only injury.

## Discussion

Non-operative management of stable thoracolumbar spinal fractures has been advocated even in the presence of kyphotic collapse (Chow et al. 1996; Shen et al. 2001; Cantor et al. 1993). Our results show that correction of these fractures offers significant benefit to patients in terms of ODI scores, return to work, time spent in hospital and secondary complications. If the spine is left with a deformity after fracture, then this has significant effects on spinal balance and the vertebral levels above and below (Xiang-Wang et al. 2008; Shen and Shen 1999). The older the patient, the less able the spine will be to compensate, and even in younger patients one would expect the spine to decompensate in later years with consequent disability. We believe that fractured spines should be treated like any other bony injury – i.e. to reduce, hold and rehabilitate, and that an unreduced deformed fracture is likely to cause disability.

Traditional open operations on the spine do lead to a legacy of soft tissue damage, particularly from stripping the posterior paraspinal muscles away from the spine (MacNab et al. 1977). A fusion adds an additional mechanical insult by creating a permanent stiff segment with stress transfer to other levels. If no fusion is carried out, then damage to those paraspinal muscles results in functional loss as those muscle groups are required to support and move those segments (MacNab et al. 1977; Choll 2010). Many surgeons try to limit the soft tissue damage caused by restricting the number of segments spanned by any construct to one level above and below the fractured vertebra. Short segment fixations such as these from the posterior aspect alone have a significant incidence of loss of correction and metalwork failure (Xiang-Wang et al. 2008; Alanay et al. 2001; Tezeren and Kuru 2005). Anterior procedures provide greater structural support to short segment posterior constructs such as these, but carry a significant complication risk and morbidity (Kim et al. 2009). Anterior procedures for thoracolumbar fractures also means taking down the diaphragm and violating the chest, which is best avoided if possible, particularly if patients have concomitant chest trauma. Stabilising the spine in these patients however has marked benefits for the recovery from associated trauma to other organs (Bellabarba et al. 2010).

MIS offers the benefits of longer segment posterior correction and fixation without the damage to soft tissues and paraspinal muscles that traditional open surgery involves (Choll 2010; Hatta et al. 2009; Smith et al. 2010). The fractured segment is stable once healed (Lindsey et al. 1993; Wang et al. 2006) and allows secondary removal of metalwork to remobilise those segments spanned (Kim et al. 2011). MIS techniques allow this without further soft tissue trauma. MIS techniques must satisfy certain criteria if they are to show benefit. There must be no compromise when using these techniques, and the surgeon must be able to achieve everything that would be attained with open surgery. Implants must be able to be placed as reliably and accurately as with open techniques. Fractures and deformity must be able to be reduced as well and reliably as with open techniques. The desired outcome should be achieved as well as with open techniques. Our study demonstrates that our techniques achieve this, and that our techniques are safe, reliable and reproducible.

Another issue is equipment and its availability. There are now a number of different systems available on the market for MIS techniques, but virtually all rely on tubes attached to the pedicle screws to guide and seat the rods allowing reduction into the pedicle screw. These systems all have a disadvantage, because they can only be used with polyaxial screws to allow for pedicle screw angle variation. This is because a tube attached to the screw head magnifies this variation, and polyaxiality is therefore required to align the screws/tubes to allow passage of the rod. This means that with these systems, the pedicle screw itself cannot be used as a vehicle to reduce a fracture or deformity. Our techniques do not have this disadvantage, because we have adapted a system designed for open surgery directly for MIS techniques. The reduction is carried out directly into the screw head on the surface of the bone, which means that we can use monoaxial or solid screws. When the reduction clamps are applied, this allows the unit to behave like a Schanz pin/screw, thereby permitting strong reliable active correction of the fracture and deformity with the pedicle screw.

Our conservative methods of treatment were standard and part of an agreed departmental protocol. Patients were assessed for brace tolerance, pressure areas and compliance (although all patients assured us they were complying), psychological factors (especially with bed rest) and problems with immobility (DVT/PE, pressure areas, bowel & bladder habit, chest problems). We have an aggressive physiotherapy protocol with regular rolling, in bed exercises & chest protocols. These protocols are comprehensive and regularly reviewed to ensure best practice.

There was a clear difference in return to work between open and conservative groups in our paper in contrast to the paper by Wood et al. In their paper, they found no difference in return to work between surgical and conservative methods (Wood et al. 2003). All of our patients who underwent MIS returned to work. This may be related to our young patient cohort. The mean ages of our patients has been stated whereas solely an age range of 18–66 years was mentioned in the paper of Wood et al.

Additionally, the paper by Wood et al. does not have the degree of kyphosis that our patients did. In their paper, the average degree of kyphosis pre-op was 10° and 5° post- surgery (Wood et al. 2003). Most surgeons would accept a pre-op kyphosis post injury of 10° but this is patient dependent. This also may indicate less violence in their patients, more stable fractures, and that the degree of kyphosis plays more of a part than their paper might perhaps gives credit to.

One would expect that for their cohort of patients with such a low degree of deformity that operative treatment would not be expected to confer an advantage. The patients in our study, by contrast, represent an entirely different group. In relation to this, the ODI scores in their paper was 20.75 at final follow up in the operative group, and 10.7 for the conservative group (Wood et al. 2003). Our ODI scores are much lower for our MIS group at 4 points, and higher for our conservative group at 32 points, which may reflect the greater degree of violence involved and the effects of a greater degree of post-traumatic kyphosis. Our MCID scores were smaller than previous studies for the ODI due to narrow standard deviations (Copay et al. 2008; Ostelo et al. 2008; Hagg et al. 2003).

We accept that the limitations of our study include the lack of variability in patients within the cohort. We have adopted strict inclusion criteria to try and make patient groups as comparable as possible. Patients were not randomised to treatment but were given the option after full discussion of the risks and benefits with the senior author of treatment options of surgery and conservative treatment. Additionally this is a study of short term follow up and longer term outcome data is required to assess the long term sequelae of such injuries.

## Conclusions

To date there has been no study to directly compare MIS techniques with open techniques using the exactly the same equipment for each. Our non-randomised, comparative study conclusively addresses this, and shows the benefits of MIS techniques. The advantages of correcting spinal fractures with a significant deformity over conservative methods where that deformity is left is evident. We would, therefore, advocate that these fractures are corrected via MIS techniques, as described above.

## Patients and methods

We have been prospectively collecting data on all single level thoracolumbar burst fractures since January 2003 to 2012.

**Figure 2:**
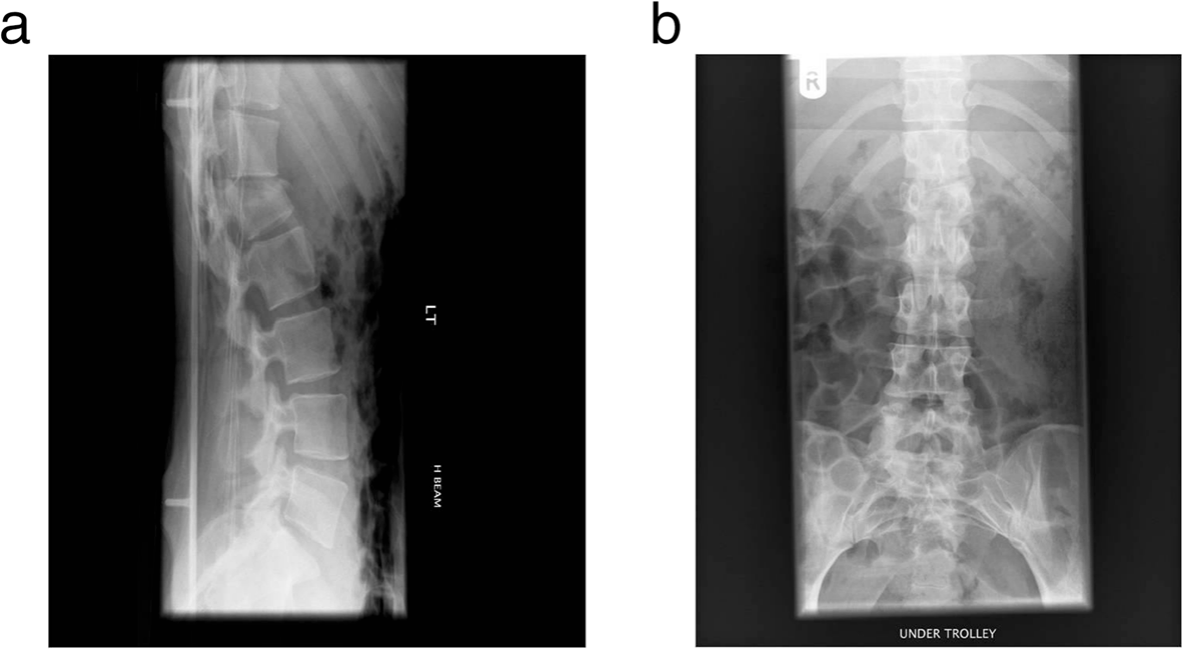
Radiograph showing a single level L1 fracture at time of injury **a**. Lateral **b**. Antero-posterior.

All fractures in the study were single level and involved a degree of local kyphosis of 20° or greater. All fractures were at either T12, L1 or L2 vertebrae and were of Magerl classification type A or type B grade (Magerl et al. 1994) without any evidence of neurological compromise (Figure 2a & b).

Additional inclusion criteria were patients of working age and in employment or full time education. All patients had no history of any back or spinal complaint and had no prior spinal surgery. Of the several groups, 30% of patients smoked and 5 % had diabetes. None had osteoporosis or any other medical condition that would affect their outcome. They were all asked to fill out an Oswestry Disability Index (ODI) form regarding their pre-accident spinal history upon admission to confirm this. All patients underwent an MRI scan at time of admission to ensure there was no significant spinal pathology elsewhere. All patients were neurologically normal clinically and radiologically. All patients had their spinal injury as their only injury. This was to ensure that when assessing patients there would be no confounding variables from other injuries that would skew or affect the results. There were, therefore, no multiply injured patients in the study.

On admission, patients were informed of the treatment options along with risks by the senior author. Patients who opted for conservative treatment were either treated with bed rest for up to 3 months if their spinal injury was deemed to be unstable, followed by TLSO bracing for 3 months; or if their injury was deemed to be stable, by TLSO bracing alone for 3 months. Stability was assessed by a MRI and CT scan of the spine along with standing radiographs in the brace. All patients underwent a functional rehabilitation programme for at least 1 year after injury.

**Figure 3:**
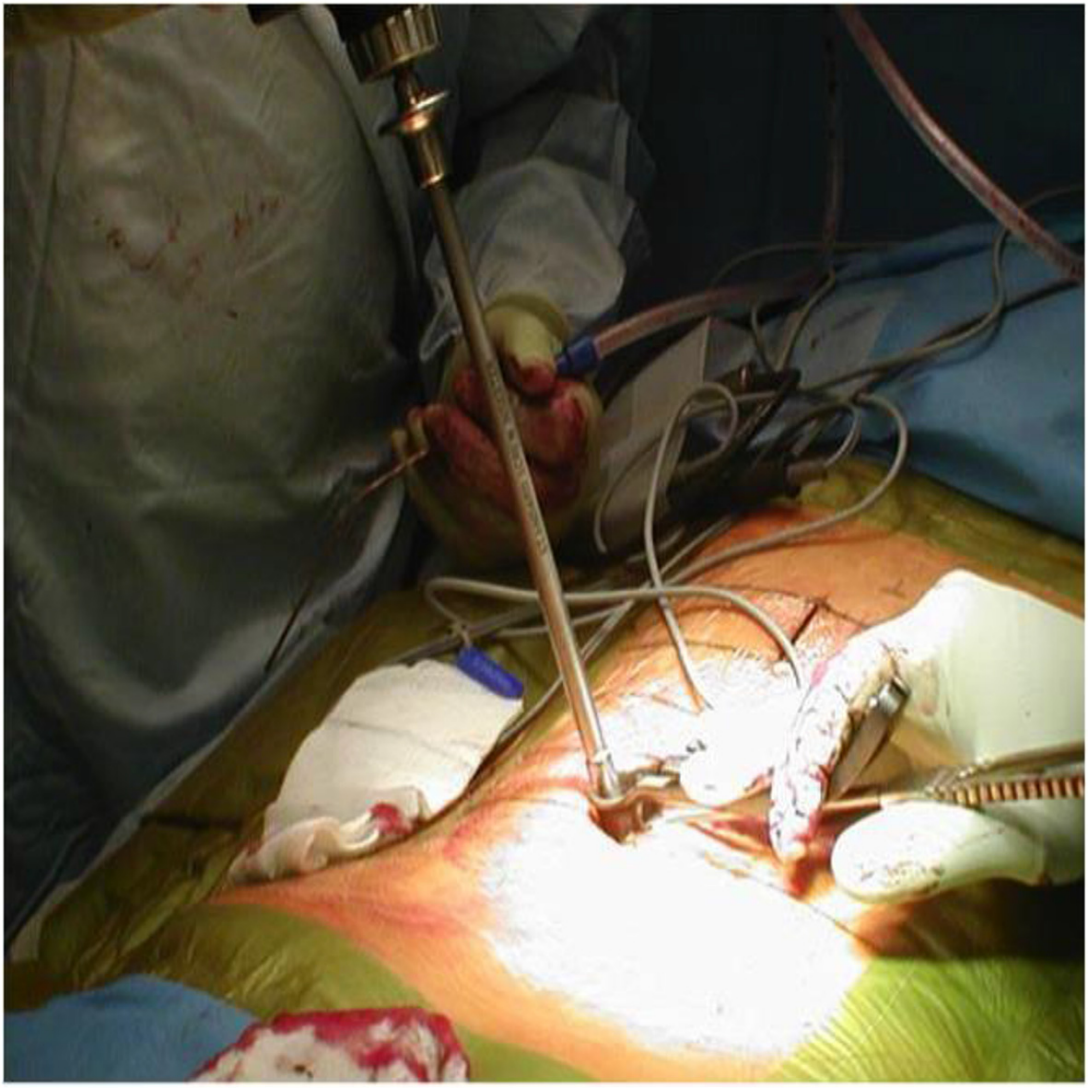
Clinical photo showing pedicle screw insertion through minimal skin incision.

**Figure 4:**
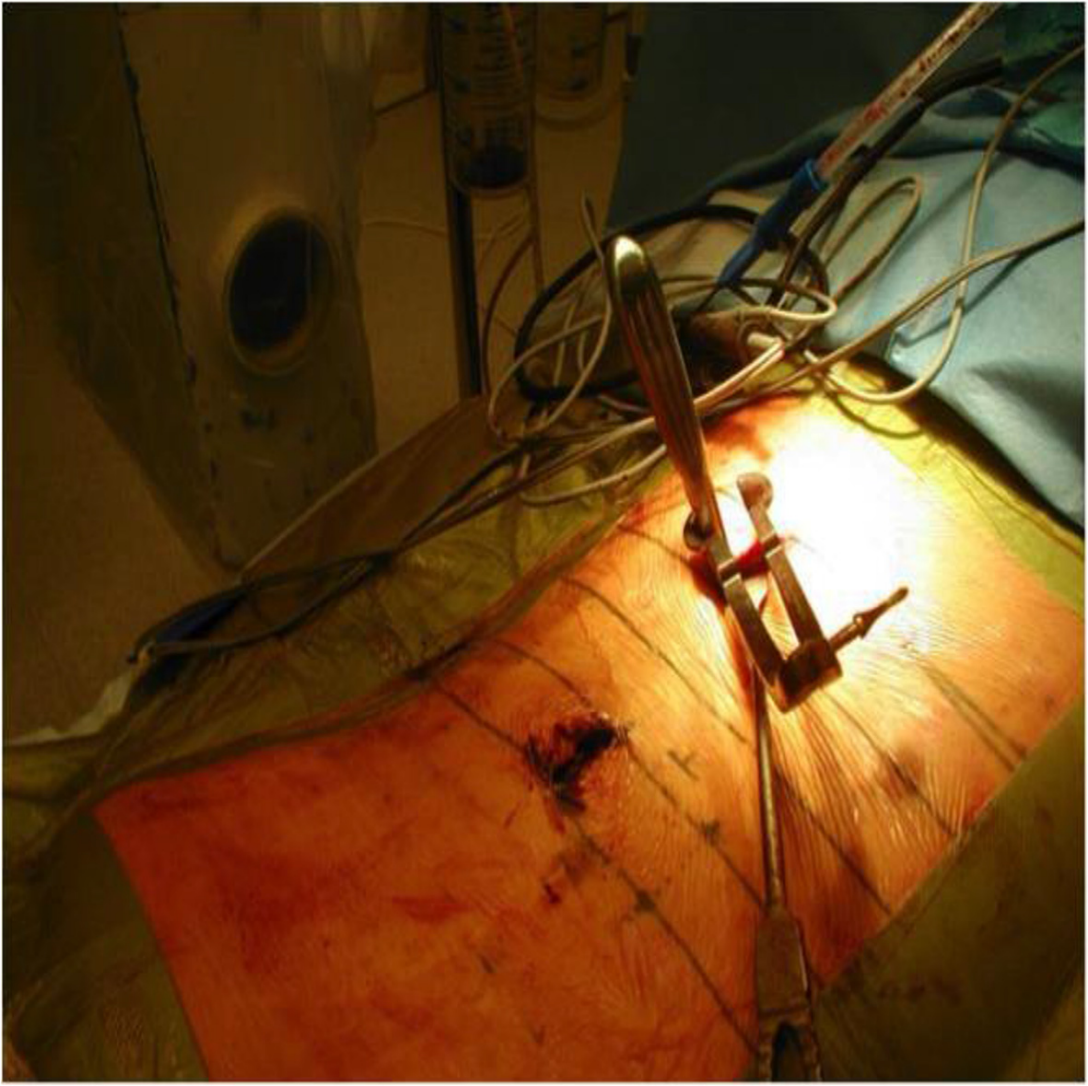
Clinical photo showing pedicle screw finder insertion through minimally invasive techniques.

**Figure 5:**
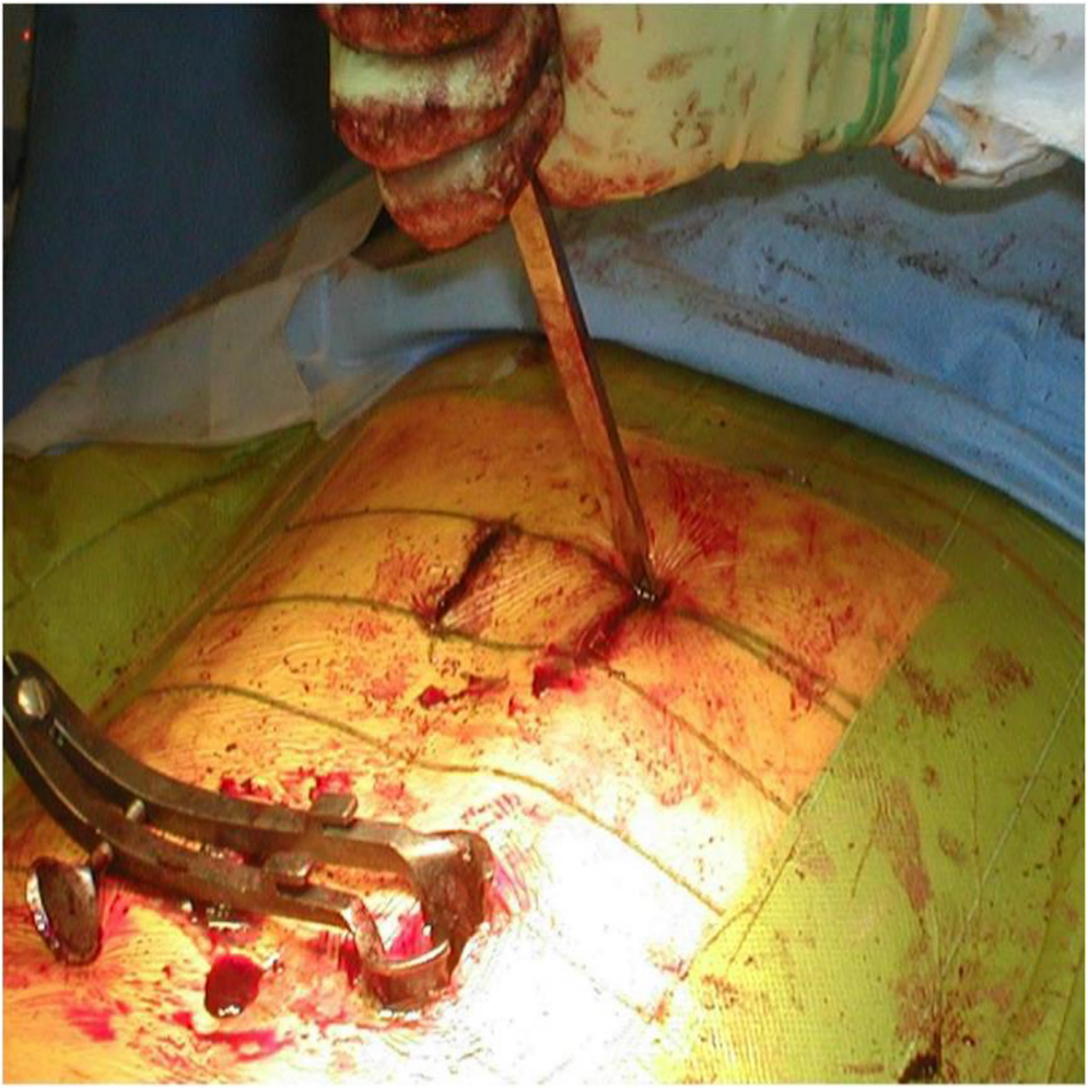
Clinical photo showing insertion of rod through small incisions.

**Figure 6:**
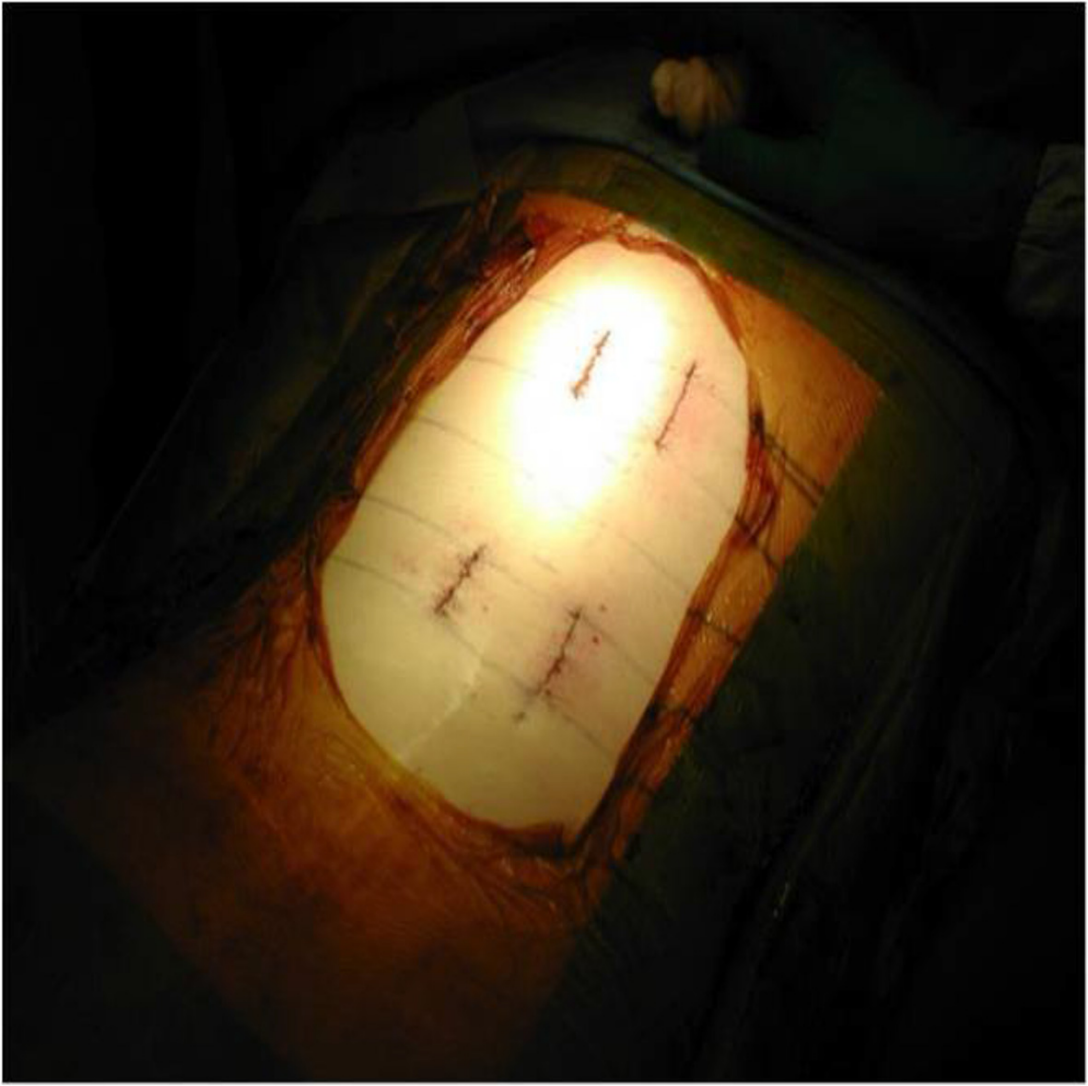
Clinical photo showing extent of MIS exposure.

**Figure 7:**
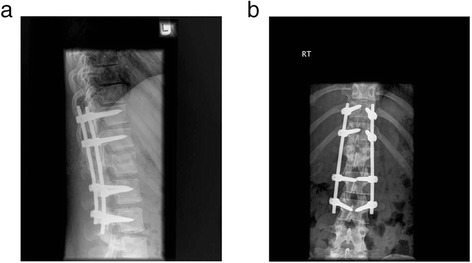
Radiograph showing stabilisation and degree of kyphosis correction **a**. Lateral **b**. Anterio-posterior.

Patients who opted for surgery were treated by open techniques until end of 2006, and via MIS techniques from 2007 until the present day. All fractures were fixed by a construct of pedicle screws inserted into the vertebrae at the 2 levels above the fractured vertebra and the 2 levels below it (Figures 3, 4, 5 and 6) (Logroscino et al. 2009; Tezeren and Kuru 2005). Decompression was not required and there was no instrumentation into the fractured vertebra itself, with no transpedicular grafting (Alanay et al. 2001). No fusion was attempted across any part of the construct as treatment was aimed for fracture correction and not fusion (Kim et al. 2011; Tezeren et al. 2009; Jindal et al. 2012). Additionally fusion would add to the operative time, blood loss and morbidity and ultimately prevent remobilisation of the spinal segment. All fractured vertebrae were corrected to less than 10° of residual kyphosis and the instrumentation used was the same for both open and MIS techniques. We used the Camlok S-Rad 90 system (Stryker GmBh) for all cases, with mono-axial (solid) screws throughout in both open and MIS cases (Figures 7).

**Figure 8:**
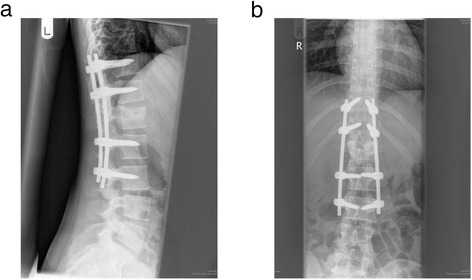
Radiograph of lower thoracic and lumbar vertebrae 12 months after stabilisation and correction **a**. Lateral **b**. Anterio-posterior.

All surgically treated patients were mobilised immediately post-operatively without any secondary bracing, and were monitored regularly post-operatively. After the removal of sutures at 2 weeks, patients were seen at 3 and 6 months post operatively with standing radiographs prior to implant removal. As this was a corrective procedure for sagittal mal-alignment not involving fusion, all implants were removed between 6 months and 1 year after surgery to remobilise the stabilised segments once the fractured vertebra had healed (Kim et al. 2011; Tezeren et al. 2009; Jindal et al. 2012) (Figure 8). Implant removal was achieved via MIS methods. If the patient had undergone open surgery, then although the old scar was opened, the implants were removed by muscle splitting portholes, the rods being slid out from underneath the muscles from the top portholes, to spare the muscles further violation from a midline approach. If the patient had had MIS techniques, then the old incisions were used and the implants were removed via the same muscle sparing techniques as above. This ensured that there was no morbidity or further trauma caused to the paraspinal muscles by implant removal.

Patients were subsequently assessed for length of stay in hospital, and time to discharge post-surgery. All patients were assessed regarding return to work status, and return to sporting or leisure pursuits. Complications, loss of correction of deformity, and the need for secondary procedures were also recorded.

All patients were followed up 1–2 weeks post injury, 6 weeks and 3 months post discharge with standing radiographs of the spine. All patients filled out an ODI assessment at follow up 18 months post-injury (i.e. at least 6 months after implant removal in those who had had surgery) and another ODI a further year later (30 months post-injury). Patients underwent standing radiographs finally at 6 months post hardware removal.

All post-treatment radiographs were analysed by the senior author (CL). Statistical analysis was performed and analysed independently on SPSS V8.0 software for windows (SPSS inc, Chicago, Illinois). Mann–Whitney U and unpaired t-tests were used to assess differences between the groups. Minimally clinically important differences (MCID) were calculated using distribution based methods involving half of the standard deviation.

Our study had local ethical approval in line with our research and audit department and as set out by the National Institution for Health Research (NIHR). Patients followed an appropriate consent procedure for their inclusion in the study.
